# A Note from Guest Editors

**Published:** 2023-12

**Authors:** Draženka Komes, Ivana Rumora Samarin

**Affiliations:** University of Zagreb, Faculty of Food Technology and Biotechnology, Pierottijeva 6, 10 000 Zagreb, Croatia

The 10^th^ Anniversary International Congress of Food Technologists, Biotechnologists and Nutritionists took place in Zagreb from 30 November to 2 December 2022 under the motto ’Smart Food for a Healthy Planet and Human Prosperity‘.

About 250 participants, including those who joined online, attended the congress. The organizers of the congress were the Croatian Society of Food Technologists, Biotechnologists and Nutritionists (PBN), the University of Zagreb Faculty of Food Biotechnology (PBF), EFFoST - the European Federation for Food Science and Technology and EHEDG - the European Association for Hygienic Engineering and Design.

The congress covered all current topics of modern society in the field of food technology, biotechnology and nutrition, which were presented in six sections: Innovation, Safety, Sustainability, Industry 4.0, Nutrition, Health and Consumer and Smart Cro.

Fifteen invited and 58 contributed speakers gave lectures, there were 146 poster presentations, and the Congress Proceedings contained 18 papers.

The congress program also included the Smart Cro section, which showcased successful start-ups from the food industry and encouraged award-winning students to present their achievements to the scientific and professional community. Parallel sections included the Biotechnology in Croatia satellite symposium ’Vera Johanides‘, presentation of the EQVEGAN project to develop the skills of future professionals in the production of the increasingly popular vegan food, and a round table on hygienic design of equipment in the food industry.

An overview of the topics of the papers shows that the focus of the researchers comprises valorisation of agri-food waste and byproducts, the application of innovative extraction techniques, plant-based food ingredients, the design of different encapsulation systems, the importance of process analytical technology (PAT) in the industry, the application of Industry 4 and digitalisation in the food industry as well as the development of smart factories.

Based on this, it is evident that the challenges of modern society are being addressed by the scientific community, giving importance to the strengthening of the collaboration of science and industry as a prerequisite for adequate responses to all future challenges.

Through its agenda, the 10th International Congress of Food Technologists, Biotechnologists and Nutritionists has created new connections and collaboration between stakeholders in research and innovation in the fields of food technology, biotechnology and nutrition, which could be a driving force for the transfer of knowledge into economic activity.

This issue of the journal contains six papers presented at the 10th International Congress and selected after a rigorous peer-review process. They address circular economy approach, improving plant food quality through controlled nutrition and enhancing the functionality of foods for special medical purposes by implementing specific probiotic strains, the chemical characterisation and antioxidant potential of autochthonous underutilised rowan fruits, the extraction of xylooligosaccharides from soybean hulls and traditional culinary plants as potential cytotoxic agents against brain tumours as an insight into the exciting discussions at the conference.

We would like to thank all the authors for their contribution to this issue and the reviewers for their insightful comments, which helped to improve the quality of the papers included in this issue.

Our thanks go to everyone who contributed in any way to the successful running of the congress and the publication of this issue.

Guest editors:

Draženka Komes, full professor

President of the Scientific Programme Committee

Ivana Rumora Samarin, associate professor

